# Establishing a model of middle cerebral artery occlusion in rabbits using endovascular interventional techniques

**DOI:** 10.3892/etm.2013.1248

**Published:** 2013-08-05

**Authors:** LEI FENG, JUN LIU, JIAN CHEN, LI PAN, GUANG FENG

**Affiliations:** 1 Department of Neurosurgery, Jining First People’s Hospital, Jining, Shandong 272011;; 2 Department of Neurosurgery, Xinqiao Hospital, The Third Military Medical University, Chongqing 400037;; 3 Department of Neurosurgery, Wuhan General Hospital of Guangzhou Military, Wuhan, Hubei 430070;; 4 Department of Neurosurgery, Changsha Eighth People’s Hospital, Changsha, Hunan 410100, P.R. China

**Keywords:** acute cerebral infarction model, rabbit, digital subtraction, diffusion magnetic resonance imaging, animal model

## Abstract

This study aimed to establish a minimally invasive and easily controllable focal cerebral ischemia model in rabbits using interventional techniques for use in the study of thrombolytic treatment, and to evaluate the feasibility and reproducibility of the technique. Under the guidance of digital subtraction angiography (DSA), focal cerebral infarction was produced by blocking the middle cerebral artery with arterial emboli to establish a rabbit brain artery occlusion model. DSA and diffusion magnetic resonance imaging (MRI) were used to observe the cerebral vascular obstruction infarction, while modified Bederson scoring was used to evaluate the neurological impairment. The animals were sacrificed 24 h after surgery and brain tissues were stained with 2,3,5-triphenyltetrazolium chloride (TTC) to evaluate the occlusion of the middle cerebral artery and pathological changes. The rabbit brain artery occlusion models were successfully established and the animals survived following embolization. Cerebral infarctions were observed in the brains of all animal models. The focal cerebral infarction rabbit model established by vascular interventional techniques is simple, minimally invasive and reliable, and may be used for early diagnosis of cerebral infarction and clinical thrombolysis studies.

## Introduction

Cerebrovascular disease, due to its high incidence, morbidity and mortality, is one of the three most serious human diseases (1–3). Ischemic stroke accounted for 70–90% of cerebral stroke cases and middle cerebral artery thromboembolism is the main cause of ischemic stroke (4,5). An in-depth study of ischemic cerebrovascular disease may useful for the prevention, treatment and prognosis of the disease. The aim of experimental studies of ischemic stroke is to establish animal models that are similar to human cerebral ischemia. Currently, methods of physically blocking blood flow by craniotomy, suture-occlusion and microembolism are used to establish animal models of cerebral ischemia (6–8). As animal models of focal cerebral ischemia are usually prepared without direct visualization, it is often not possible to accurately determine whether vascular occlusion is affected by the experimental strain, batch or weight of the animal, the proficiency of the operator or other factors. These methods have certain limitations, including poor experimental controllability and high animal mortality, which render them unsuitable for studies of thrombolytic therapy. Therefore, the establishment of an easily controllable, stable, reliable and reproducible focal cerebral ischemia model with minimal trauma to the animals, which is similar to human brain ischemia, is essential for the study of the pathophysiology, pathogenesis, prevention and control of cerebral ischemia. In the present study, an animal model of cerebral infarction was established using neurovascular interventional techniques. The technical feasibility and stability of the model have been summarized to assess the value of its clinical application.

## Material and methods

### Animals

Ten healthy male New Zealand rabbits, weighing 2.5–3.0 kg, were used in the study. This study was carried out in strict accordance with the recommendations in the Guide for the Care and Use of Laboratory Animals of the National Institutes of Health (9). The animal use protocol was reviewed and approved by the Institutional Animal Care and Use Committee (IACUC) of Jining First People’s Hospital, Jining, China.

### Thrombus preparation

The New Zealand rabbits were anesthetized with 3% sodium pentobarbital (1 ml/kg, i.v.). Following iodinated disinfection of the back of the rabbit ear, rabbit auricular endarteria were punctured and scraped with a modified lumbar puncture needle to form abrasions of ∼2 cm in the endarterium. A silk suture was tied loosely around the blood vessel to reduce blood flow, retard blood flow velocity and increase the likelihood of embolus formation. The rabbits were anesthetized again 24 h later and the scraped auricular artery was removed. The intravascular thrombus was stripped under a magnifying glass and was cut with an ophthalmic scalpel into 0.5×0.4 mm samples. The samples were placed in sterile normal saline ready for use.

### Establishing the experimental embolism model

The rabbit from which the intravascular thrombosis was stripped was anesthetized and placed supine on the operating table of the bed of the subtraction angiography machine (OEC 9800; GE Healthcare, Salt Lake City, UT, USA). The limbs were fixed, routine disinfection was performed, the right vastus medialis skin was incised and the right femoral artery was separated for preparation of intubation and thrombolysis. After the distal end of the right femoral artery was ligated and the proximal end of the artery was clipped with a temporary occlusion clip, half of the right femoral artery diameter was removed using ophthalmic scissors and a 4F arterial sheath (Terumo, Tokyo, Japan) was placed via right femoral artery puncture. Under the guidance of a TV monitor and ancillary micro-guide wire, the Echelon-10 microcatheter system (eV3, Micro Therapeutics, Inc., Irvine, CA, USA) was inserted via the right femoral artery into the right or left common carotid artery. Iodixanol (Jiangsu Hengrui Medicine Co., Ltd., Lianyungong, China) was used as the contrast agent in a ratio of 2:1 to normal saline. The catheter tip was placed flush with the lower edge of the second cervical vertebra and the lateral roadmap was made. The internal carotid artery was considered as a backward-running branch of carotid artery, with an obvious sign of ampulla-like enlargement. The micro-catheter crossed the occipital artery opening at the proximal end of the carotid artery and lateral angiography was conducted to assess the blood vessels. After the contrast agent was administered by hand to demonstrate the absence of evident reflux, three blood clot samples were injected with a 1 ml syringe through the micro-catheter. After occlusion of the middle cerebral artery was demonstrated by angiography, the micro-catheter was withdrawn. The temperature was maintained at ∼37°C after embolization and the vessel recanalization was reviewed using angiography at 2 and 5 h, respectively, after embolization. The catheter was flushed with heparin saline throughout the entire process.

### Cerebral angiography

The contrast agent iodixanol (100 mg/ml) was injected into the vessel with a high-pressure syringe bolus through the Echelon-10 micro-catheter in the internal carotid artery prior to and at 0, 2 and 5 h after embolization in each rabbit. Digital subtraction angiography (DSA) (pressure, 50 psi; speed, 0.5 ml/sec) was conducted to observe vascular thrombosis.

### Magnetic resonance imaging (MRI) examination

Diffusion weighted imaging (DWI), T_1_WI and T_2_WI scans were performed using a GE Signa HDe 1.5 T superconducting MRI scanning machine (GE Healthcare) at 2 and 5 h after successful establishment of the animal models. A small knee coil was used. The DWI parameters were as follows: echo planner imaging (EPI) list, repetition time (TR) 5,000 msec, echo time (TE) 10 msec, thickness 3 mm, interval 1 mm, field of vision (FOV) 16×16 cm, matrix 128×128 and scanning time 40 sec. The T_2_WI parameters were as follows: TR 2,500 msec, TE 99 msec, thickness 3 mm, interval 1 mm, FOV 24×24 cm and matrix 128×128. The T_1_WI parameters were as follows: TR 564 msec, TE 15 msec, thickness 3 mm, interval 1 mm, FOV 24×24 cm and matrix 128×128.

### Scoring physical signs of neurological deficits

The modified Bederson scoring (10)was used to evaluate the neurological deficits 24 h after the anesthetized animals regained consciousness. The scores were: 0, no neurologic deficit symptoms; 1, buckling of the contralateral forelimb; 2, grip of the contralateral fore-limb decreased when the tail was pulled; 3, movement without clear direction and turning in circles towards the contralateral side when the tail was grasped; 4, spontaneously turning in circles to the contralateral side. The higher the score, the more serious the neurological disorder was.

### Pathological examination

The animals were sacrificed by carotid phlebotomy 24 h after embolization. The brain tissue was completely separated from the medulla oblongata. The brain was quickly removed and placed in the freezer. When frozen for ∼20 min, the brain was cut into six coronal brain slices of ∼5 mm in thickness. The slices were checked for bleeding and incubated in 2% 2,3,5-triphenyltetrazolium chloride (TTC; Sigma, St. Louis, MO, USA) at 37°C for 30 min. The slices were then transferred into 10% formalin solution for fixation. The ischemic range of brain tissue was observed after one week. The percentage of infarction area of each rabbit was calculated using the following formula: Total infarction area/hemispheric infarction zone ×100. After the sample was hematoxylin and eosin (H&E)-stained and paraffin-embedded, histological evaluation of the sample was conducted under a light microscope.

## Results

### Signs of neurological deficits

All animals survived for 24 h after embolization. Different degrees of paralysis were observed in the limb contralateral to the embolization, including collapsing to the contralateral side, hindlimb abduction, weakness, muscle tension reduction and retractile reaction weakening. There were eight cases of hemiplegia (collapsing to the hemiplegic contralateral side or circling) and two cases of weakness of the contralateral forelimb to embolism.

### TTC staining

**Figure 1.**
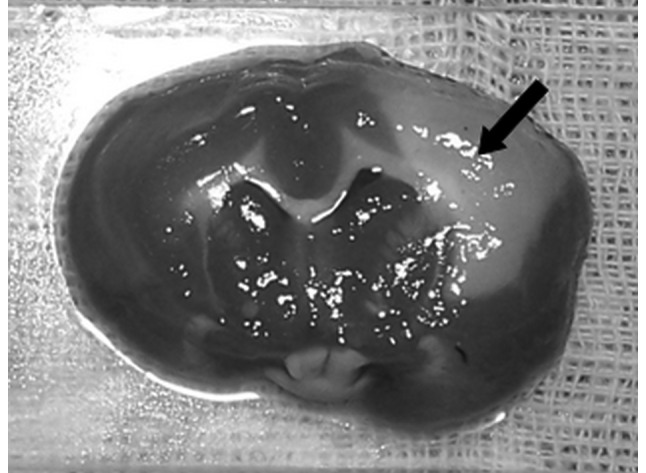
Cerebral infarction with TTC staining. Off-white area is the infarcted zone. TTC, 2,3,5-triphenyltetrazolium chloride.

The infarction was clearly visible in the distribution of the ipsilateral middle cerebral artery 24 h after embolism. It was also possible to observe an irregular pattern of infarction with the naked eye. This ranged in size from 0.5 to 2.0 cm with a gray central region and clear boundary (Fig. 1). The percentage of infarction area was 38.67±2.23%.

### Cerebral angiography

**Figure 2.**
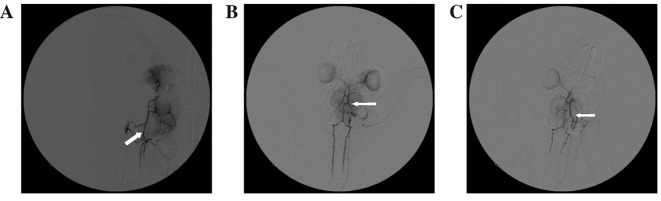
(A) Rabbit normal cerebrovascular lateral DSA. The occipital artery is indicated by the arrow. (B) Rabbit normal cerebrovascular anteroposterior DSA. The middle cerebral artery is indicated by the arrow. (C) DSA of the rabbit brain 5 h after the model was established. The brain arteries without development are indicated by the arrow. DSA, digital subtraction angiography.

Part of middle cerebral artery branches did not develop after the injection of autologous blood clots in the internal carotid artery (Fig. 2). The results of angiography showed that no recanalization occurred in the clogged vessels at 2 or 5 h after embolization.

### MRI examination

**Figure 3.**
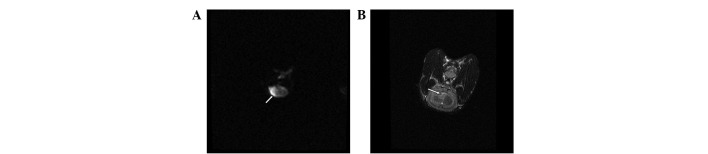
(A) DWI captured 5 h after the model was established. The arrow shows the infarction. (B) T_2_ MRI 5 h after the model was established. The arrow shows cerebral infarction. DWI, diffusion weighted images; MRI, magnetic resonance imaging.

T_2_WI was negative when ischemia had been induced for 2 h in the ten rabbits. After 5 h of induced ischemia, there was a higher sign or greater signal change in all ten rabbits (Fig. 3).

### Light microscopic examination

**Figure 4.**
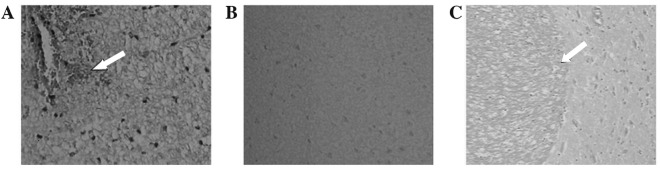
Pathological results under a light microscope (magnification, ×200). (A) A small amount of exudative bleeding was observed. The white arrowhead indicates cell swelling, nuclear condensation and disappearance. (B) Large amounts of neurons and mesh-like cells in vacuolar changes are visible. (C) Normal brain tissue and infarcted area are clear and legible (large black arrow).

The results of examination by light microscopy showed that the brain tissues contralateral to the embolization stained by H&E were normal. Observation of the embolization under a light microscope indicated that edema was present in the brain tissue of the infarct area and coagulative necrosis occurred widely in the nerve cells. The nuclei became pyknotic or disappeared and the cell outline was vague. The nerve and glial cells were significantly reduced or disappeared. The neutrophil infiltration occurred in a discrete area. Microglia hyperplasia and neurotropic phenomena were also observed. The boundary between the infarction and the normal area was clear and an infarcted area, zone of edema and normal area were visible from the infarction outwards (Fig. 4).

## Discussion

Prior to an experimental study of the thrombolytic treatment of acute cerebral infarction, an ideal animal model of cerebral ischemia should first be established. Focal ischemic cerebrovascular diseases are common in the clinic, particularly middle cerebral artery occlusion. Due to poor collateral circulation, the middle cerebral artery occlusion model has been recognized as the standard animal model of focal cerebral infarction (11). The experimental results in the current study indicated that the method used for establishing the animal model is reliable and repeatable. The process used to generate the model is similar to that by which human ischemic stroke occurs and the cerebral blood flow is easily observed. The model established by this method is conducive to the observation of the subsequent effect of thrombolysis and is applicable to neuroimaging research on cerebral ischemia.

Common methods for creating models of focal cerebral ischemia include opening the skull to physically block the middle cerebral artery with electric coagulation (12), suture-occlusion (13,14) and microembolism (6,7). As these animal models are different from human stroke, they are not suitable for use in studies of thrombolysis and anticoagulation therapy. An animal model of focal cerebral infarction caused by autologous thromboembolism is similar to the pathological process of human ischemic stroke and therefore has a certain value for the evaluation of thrombolytic or anticoagulant efficacy (15–19). The ideal animal model of acute brain artery occlusion used for superselective intra-arterial thrombolysis should meet the following requirements: i) The animal vascular anatomy should be close to that of human brain blood vessels, and cerebral angiography and superselective intra-arterial thrombolysis should be relatively easy to perform. ii) Cerebrovascular variation of the animal should be minimal, with the location and extent of the embolism being fixed readily and with good reproducibility. iii) Success and animal survival rates should be high, with minimal surgical injury. iv) Observation of vascular occlusion should be possible from the changes in the blood flow. v) The animal blood should be sufficient to be drawn repeatedly and be suitable for dynamic observation of certain biochemical changes. Embolization in the rabbit middle cerebral artery infarction under DSA may make the control of the infarction conditions relatively consistent. Rabbit blood is sufficient in quantity for repeated drawing and is also suitable for dynamic observation of certain biochemical changes. The rabbit vascular anatomy is close to that of human brain blood vessels and cerebral angiography may be easily completed. Therefore, rabbits are suitable for use as a thrombolytic animal model.

The preparation of thromboemboli is critical for the successful establishment of a model. In humans, 75% of cerebral emboli comprise ‘white thrombus’, i.e., are rich in platelets and fibrin (20). An ideal thrombosis should be rich in cellulose, with no deformation, be difficult to autolyze and of sufficient size to block the middle cerebral artery initial segment. Thrombi formed within arterial or venous blood vessels are red and the main constituents are red blood cells and platelets. The thrombi that readily undergo autolysis are subject to further processing (15,16,21–23). In the present study, autologous arterial thrombi were prepared by puncturing and scraping the rabbit auricular endarterium to form abrasions with a modified lumbar puncture needle. The thrombus formed in this way is rich in fibrin and platelets, and similar to those formed in human atherosclerosis, and so is more suitable for the study of thrombolytic therapy.

In a previous study, the embolus was injected by intubation through the internal carotid artery using the Hamilton method (24). This model may often result in injury to the internal carotid artery and readily cause the formation of thrombosis that may affect the normal flow of the internal carotid artery. In the Benes method (22), external and internal carotid arteries near the skull base are anatomized. Following the ligation of each branch of the external carotid artery, intubation was inserted retrogradely into the opening of the internal carotid artery. As it is difficult to surgically expose the bifurcation, microsurgery is often required to anatomize the carotid bifurcation. Therefore, the high technical requirement and complexity of the modeling process may result in serious animal trauma and high mortality. In addition, 25.7% of the rabbit occipital artery originates from the internal carotid artery, and 13.3% of occipital arteries with a large diameter originate from the distal end of the internal carotid artery near the skull base (25). Since the previous method may fail to ligate the occipital artery separated from the internal carotid artery, resulting in some emboli entering into the occipital artery, the success rate was only 50–85% (24). To ensure the probability of success, superselective intubation of the internal carotid artery was used for embolization of the middle cerebral artery after crossing the opening of occipital artery near the proximal end of the internal carotid artery or embolization of the occipital artery.

The results of angiography demonstrated that the internal carotid artery of the rabbit is the main blood supply of the intracranial artery and there is no anastomosis network between the intracranial and extracranial vessels. End-to-end anastomosis among branches of the brain blood vessels is similar to that of the human brain. The occipital artery originates from the internal carotid artery and its distal end may diminish in size to half that of the initial part. The distal diameter of the internal carotid artery, other than the separated occipital artery branch, was not observed to change significantly. The extracranial segment of the rabbit carotid artery is tortuous and slender to form a loop during ascent, which is similar to the human carotid siphon. Two branches are separated from its ends; following the separation of the middle and anterior cerebral arteries, some of the blood vessels originating from the former branch supply blood to the eye, head and facial organs while the latter branch, which is rearward and downward in direction, is the posterior communicating artery. The two bilateral branches are able to form the circle of Willis with the basilar artery and have no anastomosis with the external carotid artery. As the rabbit circle of Willis has good compensation, it is difficult for a large thrombus to form a cerebral infarction due to embolization in the internal carotid artery. The size of the autologous thrombus in the current study was 0.5×0.4 mm, which is not sufficient to directly block the internal carotid artery. Following the insertion of a micro-catheter into the internal carotid artery by ∼1 cm, a contrast agent may be injected and, if no regurgitation is evident, a thrombus may subsequently be injected.

Since the middle cerebral artery with its relatively large blood flow is the largest branch of the internal carotid artery, the probability of occlusion in the middle cerebral artery is the highest. The incidence of embolization in posterior communicating artery running backward and downward was the lowest, which was basically in line with the morbidity in humans. Embolization to the proximal end in M1 infarction occurs in the cerebral hemispheres and basal ganglia. Embolization to the distal end of M1 mainly manifests in the lobular cortex. Embolization to the distal end of M2 generally results in focal cerebral infarction. Since the current interventional devices are not yet able to selectively insert emboli into the rabbit middle cerebral artery, shortcomings remain. These include instability in the blocking of the vessels and the occasional entry of emboli into the anterior cerebral or contralateral intracranial artery.

DSA cerebral angiography may accurately display thromboembolic site thrombolysis and artery recanalization, but not abnormal brain parenchyma and infarct range. Functional magnetic resonance DWI may be used to determine the ischemic range. It also may be used to evaluate the efficacy of thrombolytic therapy and for the study of ischemic penumbra. In the current study, all animals were sacrificed 24 h after embolization and the results of TTC staining also demonstrated the infarction area, location and the scope in the middle cerebral artery, which were consistent with the results of examination by functional magnetic resonance DWI.

In the current study, we noted the following: i) In order to achieve good controllability, reduce surgery time and increase the success rate, the roadmap function may be used to make the operation of the catheter and guide wire visible. ii) Operation of the micro-guide wire should be performed gently. The micro-guide wire tension should be released, particularly when entering into the middle cerebral artery, so as not to pierce the blood vessel, and thereby cause cerebral hemorrhage and model failure. iii) Continuously washing with heparin saline during surgery may effectively prevent thrombosis from the cerebral infarction occurring outside the target area. This model may avoid the large trauma caused by surgery at the neck and is conducive to the animal’s rapid recovery and the long-term observation of the model, as well as the treatment effect. The disadvantage is the requirement for a subtraction angiography machine and professional personnel who are capable of carrying out the endovascular interventional technique.

In conclusion, we performed technological improvements to an experimental animal model of focal cerebral ischemia and successfully established focal cerebral ischemia animal models using endovascular interventional techniques. The results suggest that the rabbit animal model of acute cerebral embolism was successfully established using embolization techniques through superselective catheterization to the rabbit carotid artery beyond the separated occipital artery branch. This method has numerous advantages, including having no requirement for craniotomy, minimal trauma, a reliable ischemic effect and a high animal survival rate, and is particularly suitable for the study of selective intra-arterial thrombolytic therapy. The model has the following notable features: the uniformity of the embolus size, stability, similarity to clinical cerebral thrombosis and suitability for the study of thrombolytic therapy. In addition, the probability of ectopic embolism is greatly reduced using superselective embolization; small amounts of emboli may create marked symptoms of cerebral ischemia; there is minimal injury to the animal with a high success rate and low mortality; and the model embolism is similar to human cerebral embolism. The present model also has a wide range of applications with scalability and may be commonly used in other experimental animals, including dogs and monkeys. Further applications of the model are studies concerning the diagnostic imaging of cerebral infarction and pathophysiological changes. Angiography is able to accurately display the thromboembolic site thrombolysis and artery recanalization.

The embolism model established in this study is similar to human cerebral embolism, with little injury, high success rate and low mortality rate. This model may suitably expanded to application in commonly used experimental animals, including rabbits, dogs and monkeys. The establishment of this model provides a broad prospect for further study of the morphology, functional metabolomics, pharmacology, clinical therapeutics, neurological interventional radiology and neurosurgery of cerebral ischemia.
